# Protist diversity and community assembly in surface sediments of the South China Sea

**DOI:** 10.1002/mbo3.891

**Published:** 2019-06-19

**Authors:** Wenxue Wu, Bangqin Huang

**Affiliations:** ^1^ School of Marine Sciences Sun Yat‐sen University Zhuhai China; ^2^ Southern Marine Science and Engineering Guangdong Laboratory (Zhuhai) Zhuhai China; ^3^ Fujian Provincial Key Laboratory of Coastal Ecology and Environmental Studies Xiamen University Xiamen China

**Keywords:** 18S rDNA, dispersal limitation, pyrosequencing, species sorting, UniFrac distance

## Abstract

Protists are pivotal components of marine ecosystems in terms of their high diversity, but protist communities have been poorly explored in benthic environments. Here, we investigated protist diversity and community assembly in surface sediments in the South China Sea (SCS) at a basin scale. Pyrosequencing of 18S rDNA was performed for a total of six samples taken from the surface seafloor at water depths ranging from 79 to 2,939 m. We found that Cercozoa was the dominant group, accounting for an average of 39.9% and 25.3% of the reads and operational taxonomic units (OTUs), respectively. The Cercozoa taxa were highly diverse, comprising 14 phylogenetic clades, six of which were affiliated with unknown groups belonging to Filosa and Endomyxa. Fungi were also an important group in both read‐ (18.1% on average) and OTU‐derived (9.3% on average) results. Moreover, the turnover patterns of the protist communities were differently explained by species sorting (53.3%), dispersal limitation (33.3%), mass effects (0%), and drift (13.3%). In summary, our findings show that the basin‐wide protist communities in the surface sediments of the SCS are primarily dominated by Cercozoa and are mainly assembled by species sorting and dispersal limitation.

## Graphical abstract

Diversity of Cercozoa in surface sediments of the South China Sea.

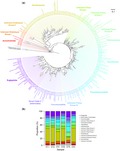

## 1 INTRODUCTION

Protists constitute essential components of marine sediment systems (Orsi, 2018). Importantly, protists play diverse roles in maintaining benthic ecosystem functioning. For example, protists exert significant influences on bacterial communities via grazing effects in deep‐sea sediments and further alter the hydrocarbon‐degrading process (Beaudoin et al., 2016). Metabolically active protists are widely detected in the subsurface of sea floors (Edgcomb, Kysela, Teske, de Vera Gomez, & Sogin, 2002), in which protists maintain important biogeochemical cycles (Edgcomb et al., 2016). In addition, protists can dominate the biomass of benthic microbiomes (Bochdansky, Clouse, & Herndl, 2017) and persist at record depths (>1,500 m) below the seafloor of the Canterbury Basin (Ciobanu et al., 2014).

Protist diversity has been poorly investigated in marine sediments compared to planktonic systems (Cheung, Au, Chu, Kwan, & Wong, 2010; Christaki et al., 2014; Logares et al., 2014; Stoeck et al., 2010; de Vargas et al., 2015; Wu, Logares, Huang, & Hsieh, 2017). A few consensuses have been reached for planktonic protists, such as the dominance of parasite groups within Alveolata (Guillou et al., 2008; de Vargas et al., 2015). Moreover, it is well recognized that benthic protists are significantly different from planktonic groups (Chen, Pan, Yu, Yang, & Zhang, 2017; Cleary & Durbin, 2016; Coolen & Shtereva, 2009; Epstein & López‐García, 2008; Massana et al., 2015) and can even exhibit higher diversity than planktonic taxa (Chen et al., 2017; Forster et al., 2016). Furthermore, deep‐sea protists are much less studied (Pawlowski et al., 2011) relative to protists in coastal and shallow‐sea sediments (e.g., Gong et al., 2015; Massana et al., 2015; Chen et al., 2017).

Little is known about how protist communities are assembled in deep‐sea sediments from a metacommunity perspective (Leibold et al., 2004; Vellend, 2010). Petro, Starnawski, Schramm, and Kjeldsen (2017) proposed four major processes of microbial community assembly in marine sediments: selection (i.e., species sorting), dispersal, diversification, and drift. As the predominant process (Petro et al., 2017), species sorting may be imposed by sediment differences such as water depth, pressure, and the properties of sediment particles. Moreover, dispersal limitation (derived from low dispersal), rather than mass effects (representing high dispersal), accounts for the importance of microbial dispersal in marine sediments because the microbial dispersal is passive and largely limited at a large spatial scale (e.g., the basin scale). Diversification (i.e., speciation) is supposed to have little influence within a metacommunity with individual dispersal (Stegen et al., 2013). Drift (acting alone), resulting from stochastic changes in birth and death rates, can be the dominant mechanism in extremely uniform habitats, which is not the case in marine sediments (Jacob, Soltwedel, Boetius, & Ramette, 2013). Therefore, we hypothesized that compositional turnover in protist communities in marine sediments at a basin scale would be mainly governed by a combination of species sorting and dispersal limitation.

The South China Sea (SCS) is one of the largest marginal seas located in the western Pacific Ocean, but the protist diversity across the basin‐wide SCS sediments remains unclear. The SCS is characterized by a wide water depth range spanning over 5,000 m accompanied by distinct types of sediments (Liu et al., 2013). These sediments with contrasting characteristics have been shown to contribute to the compositional turnover in benthic microbial communities (Zhu, Tanabe, Yang, Zhang, & Sun, 2013). In addition, the semiclosed SCS is strongly influenced by the regulation of surface circulations by the East Asian monsoon system (Liu et al., 2002), which can also influence the seafloor microbial communities (Hamdan et al., 2013). This influence is partially due to seasonal monsoons that contribute to the transport of fluvial sediments in the SCS (Liu et al., 2016; Schroeder, Wiesner, & Liu, 2015).

The goal of this study was to investigate protist diversity and community assembly in surface sediments of the SCS. We investigated six sites (79–2,939 m depth) that represented common habitat types in the SCS seafloor and performed pyrosequencing of the V1–V2 region of 18S rDNA. We revealed the underlying processes that regulated community patterns of benthic protists using null model analysis and tested the hypothesis that species sorting and dispersal limitation are the two key driving forces. Overall, this study provides baseline information on the protist diversity and assembly in surface sediments of the SCS.

## 2 MATERIALS AND METHODS

### 2.1 Sample collection

**Figure 1:**
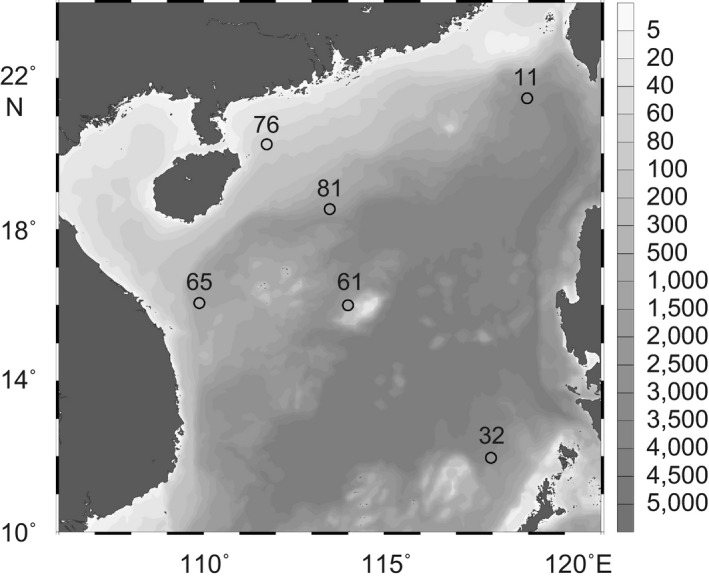
Locations of the six samples (circles) taken from surface sediments in the South China Sea. Gray contours represent bottom depths (m). The map was generated using Ocean Data View (Schlitzer, 2018)

A total of six sediment samples were collected from the surface seafloor using a grab sampler in the SCS during 28th April–21st May in 2010 (Figure 1). This sampling design included one station (ST76) from the shallow coast (water depth = 79 m) and five stations located in the deep basin (water depths >880 m) (Table 1). Surface sediment samples (0–20 cm) were immediately collected and stored at −20°C until further analyses. Hydrodynamic profiles (i.e., temperature and salinity with water depth) of the upper waters at each station were obtained with an SBE‐911 instrument (Sea‐Bird Electronics, USA).

**Table 1:** Summary of sampling information (locations and water depths), sequencing results (the number of quality‐checked reads and observed operational taxonomic units, OTUs) and richness estimators based on an equal sequencing depth of 5,792 reads (Chao1 and Shannon indexes)

Sample	Station	Depth (m)	Quality‐checked reads	OTUs	Chao1	Shannon
ST11	11	2,801	13,620	370	461	4.42
ST32	32	2,939	8,911	315	368	3.6
ST61	61	1,250	14,022	317	371	4.21
ST65	65	880	10,737	402	475	4.71
ST76	76	79	5,792	341	474	4.26
ST81	81	1,469	21,009	276	301	4.2

### 2.2 DNA extraction and pyrosequencing

For each sediment sample, the top 0–1 cm segment was used for molecular analyses. Total DNA was extracted using an UltraClean Soil DNA Isolation Kit (MO BIO Laboratories, USA) according to the manufacturer's instructions, during which samples were homogenized for 60 s at 4 m/s using a FastPrep‐24 instrument (MP Biomedicals, USA). The DNA extracts were quantified using a NanoDrop ND‐1000 spectrophotometer (Nanodrop Technologies, USA). PCR amplification was performed for the V1–V2 region of 18S rDNA (approximately 420 bp) using the primers SSU_F04 (5'‐GCTTGTCTCAAAGATTAAGCC‐3') and SSU_R22 (5'‐GCCTGCTGCCTTCCTTGGA‐3') (Bik et al., 2012). The PCR program consisted of an initial denaturation step at 95°C for 2 min; 30 cycles of 95°C for 30 s, 53°C for 30 s and 72°C for 30 s; and a final extension at 72°C for 5 min. The amplification products were then purified using an AxyPrep DNA Gel Extraction Kit (Axygen, USA). Pyrosequencing was carried out on a 454 GS FLX Titanium system (Roche, USA) following the manufacturer's instructions. Raw sequence data have been deposited in the Sequence Read Archive (NCBI) under accession number SRP083955.

### 2.3 Sequence processing

The pyrosequencing data were processed using the Quantitative Insights Into Microbial Ecology (QIIME v. 1.9.1) pipeline (Caporaso et al., 2010). Briefly, the quality of reads was checked using a 50‐bp sliding window and an average Phred threshold of 25, and short reads (<200 bp) were discarded. The remaining reads were run through DeNoiser (Reeder & Knight, 2010) to reduce pyrosequencing errors. The resulting sequences were grouped into operational taxonomic units (OTUs) using UCLUST (Edgar, 2010) with a minimum identity of 97%. The representative sequence per OTU was selected, and chimeras were checked using ChimeraSlayer (Haas et al., 2011). The assignment of the representative sequences was determined using the PR^2^ database (Guillou et al., 2013) with a BLAST *E*‐value of 10^−6^ and a minimum percent similarity of 90% (Zhang, Schwartz, Wagner, & Miller, 2000). Singletons (OTUs with only a single sequence in the entire data set) and OTUs with sequences detected in only a single sample were removed. Metazoans, as multicellular animals, were also removed because this study focused on single‐celled protists. Consequently, OTUs assigned to metazoans were removed from further analyses. OTU representative sequences were aligned using MAFFT with the FFT‐NS‐2 method (Katoh & Standley, 2013), and the resulting alignments were used to generate a phylogenetic tree with FastTree (Price, Dehal, & Arkin, 2009).

### 2.4 Phylogenetic analysis of Cercozoa

Considering the large percentage of Cercozoa sequences detected in sediment protist communities, we performed detailed phylogenetic analyses of the benthic Cercozoa. We carefully checked all representative sequences affiliated with the Cercozoa to ensure the performance of the phylogenetic analysis. The raw reads were generated from the orientation of the forward primer, while only sequences containing the accurate reverse primer (no mismatches) were retained in the subset of Cercozoa. All resulting sequences were aligned using MAFFT with the E‐INS‐i method, and the reverse primer was excluded. Each sequence was then manually checked using BLAST against the GenBank database. If a sequence had a similarity lower than 90% with the GenBank top hit and was rare (relative abundance <1% in all samples), we removed it from the data set. Reference sequences were added to perform phylogenetic analyses, and the whole sequences were aligned using the E‐INS‐i method. We manually trimmed positions with >95% gaps in each aligned column. A maximum‐likelihood phylogenetic tree was constructed using PhyML (Guindon et al., 2010) with 1,000 bootstraps and the GTR + G + I model.

### 2.5 Statistical analysis

Rarefaction analyses were performed to examine the degree of sampling saturation. To compare the OTU richness among the six sediment samples, we calculated nonparametric richness estimators (Chao1 and Shannon indexes). Chao1 and Shannon indexes were estimated based on the standardized data of 5,792 sequences per sample using the vegan package (Oksanen et al., 2014). To compare community dissimilarities, we performed phylogenetically informed beta diversity analyses using the weighted UniFrac distance metric (Lozupone & Knight, 2005) implemented in the QIIME pipeline (based on a standardized OTU table of 5,792 sequences per sample). Principal coordinates analysis (PCoA) was conducted on the weighted UniFrac distances to display the results. To further examine community dissimilarities (i.e., weighted UniFrac distance) against water depth, a Mantel test was performed using the vegan package. However, we could not rule out that other unmeasured environmental factors might also be important in shaping these benthic protist communities.

Null model analysis was performed to estimate the relative importance of different ecological processes (i.e., species sorting, dispersal limitation, mass effects, and drift) using the framework of Stegen et al. (2013). First, the between‐community variation in βMNTD was calculated based on the rarified OTU table (5,792 sequences per sample) using the picante package (Kembel et al., 2010). The degree to which the observed βMNTD deviated from a null model expectation was quantified after 999 randomizations. Standardized effect sizes of βMNTD (i.e., βNTI) <−2 or >2 indicated that compositional differences between community pairs were driven by species sorting. Second, we calculated the Raup‐Crick dissimilarity metric (RC_bray_) for each community pair (999 null iterations) for cases of |βNTI| <2. RC_bray _values >+0.95, <−0.95, and between −0.95 and +0.95 were assumed to indicate the operation of dispersal limitation, mass effects, and drift, respectively. Statistical analyses were mainly conducted in R (R Core Team, 2018).

## 3 RESULTS

### 3.1 Water column environment

**Figure A1:**
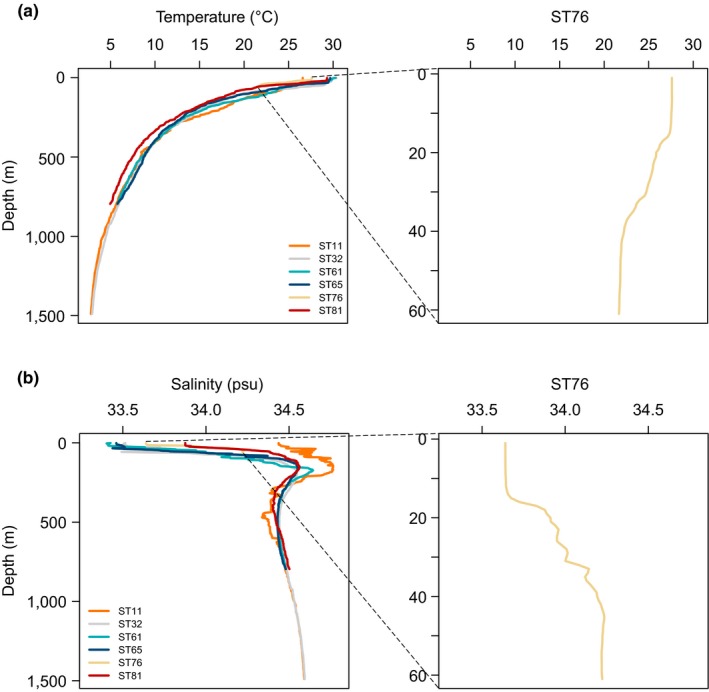
Temperature (a) and salinity (b) profiles of the upper waters of the sampling sites. Vertical profiles are based on the records at a water depth of 1,489, 1,489,795, 796, 61, and 796 m at ST11, ST32, ST61, ST65, ST76, and ST81, respectively

Vertical hydrographic profiles of the upper waters indicated that the sampling sites were characterized by low temperature (e.g., 5.8°C at a 796 m depth at ST65; Figure A1a) and high salinity (e.g., 34.5 psu at a 795 m depth at ST61; Figure A1b), except for the coastal site ST76 (21.7°C and 34.2 psu at a 61 m depth). However, detailed in situ environmental variables were unavailable for sediments.

### 3.2 Benthic diversity

**Figure 2:**
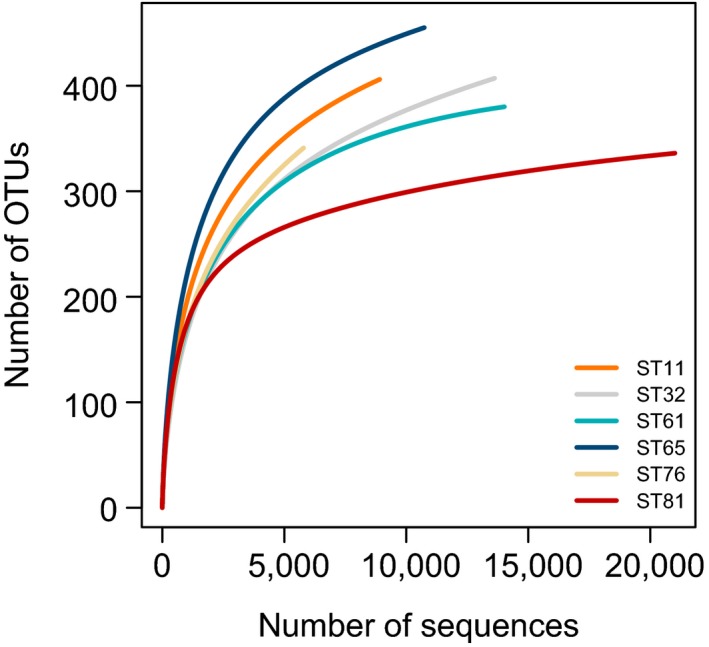
Rarefaction curves of observed OTUs for the six samples indicated by different colors. OTUs, operational taxonomic units

Pyrosequencing recovered a total of 74,091 quality‐filtered reads (5,792–21,009 reads per sample) that were grouped into 269–408 OTUs per sample (Table 1). The rarefaction curves of the observed OTUs showed unsaturated sampling profiles for all six samples (Figure 2), indicating high diversity of benthic protists. ST81 and ST65 had the lowest and highest richness, respectively, based on an equal sequencing depth of 5,792 reads (Table 1).

**Figure 3:**
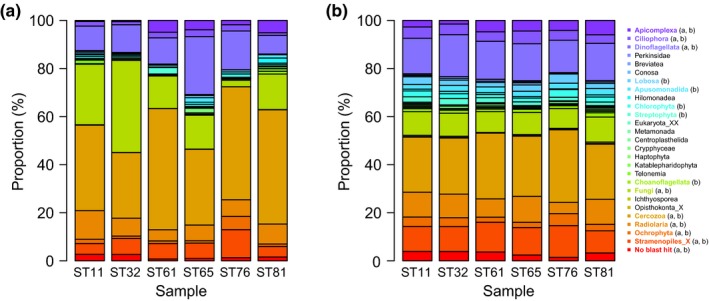
Taxonomic compositions (%) of reads (a) and OTUs (b). The groups showing an average contribution greater than 1% in the six samples are in bold and marked with corresponding colors in the bar plots, and the panel in which these groups are abundant (>1%) is indicated by the character in brackets. OTUs, operational taxonomic units

Based on the read‐based community patterns, Cercozoa was the most abundant group, accounting for proportions ranging from 27.3% (ST32) to 50.4% (ST61) (Figure 3a). Fungi were another abundant group, with an average proportion of 18.1% (Figure 3a). Remarkably, fungi comprised 38.3% of the total sequences at ST32 and were thus the most abundant group. Dinoflagellata also made substantial contributions ranging from 7.7% (ST81) to 24.1% (ST65). Radiolaria and stramenopiles_X had comparatively stable proportions across the six samples, showing an average of 7.6% and 6.7%, respectively. A few photosynthetic groups were retrieved, such as Cryptophyceae, Chlorophyta, Haptophyta, and Streptophyta. In particular, Cryptophyceae accounted for 2% of the sequences at ST65 (Figure 3a). Based on the OTU‐based community patterns, Cercozoa repeatedly appeared as the most abundant group, showing an average proportion of 25.3%, followed by Dinoflagellata (15.4%), stramenopiles_X (11.1%), and fungi (9.3%) (Figure 3b).

### 3.3 Cercozoa dominate benthic diversity

**Figure 4:**
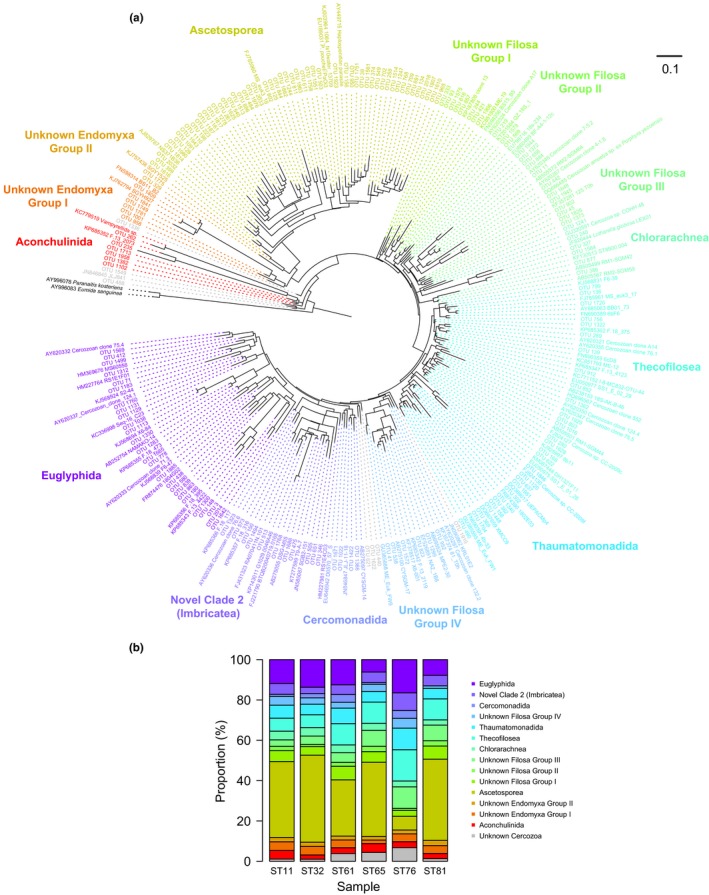
(a) Maximum‐likelihood tree inferred from 18S rDNA sequences of Cercozoa (376 positions) in surface sediments from the South China Sea. Taxa include the sequences obtained by pyrosequencing and reference sequences. Subgroups are color coded according to taxonomic assignments. The scale bar corresponds to 0.1 substitutions per base. (b) Relative contribution (%) of subclade OTUs to the total Cercozoa OTUs in each sample. OTUs, operational taxonomic units

Sequences of Cercozoa were clustered into 180 OTUs belonging to 14 phylogenetic groups (Figure 4a), suggesting a striking diversity of benthic Cercozoa. Remarkably, a few phylogenetic groups belonged to unknown clades (e.g., Unknown Filosa Groups I, II, III, and IV; Unknown Endomyxa Groups I and II), indicating that they might be novel taxa. Ascetosporea, Euglyphida, and Thecofilosea, as the top 3 groups, contributed an average proportion of 32.1%, 11.4%, and 10%, respectively, to the total Cercozoa OTUs (Figure 4b and Table A1).

### 3.4 Benthic community structure and assembly

**Figure 5:**
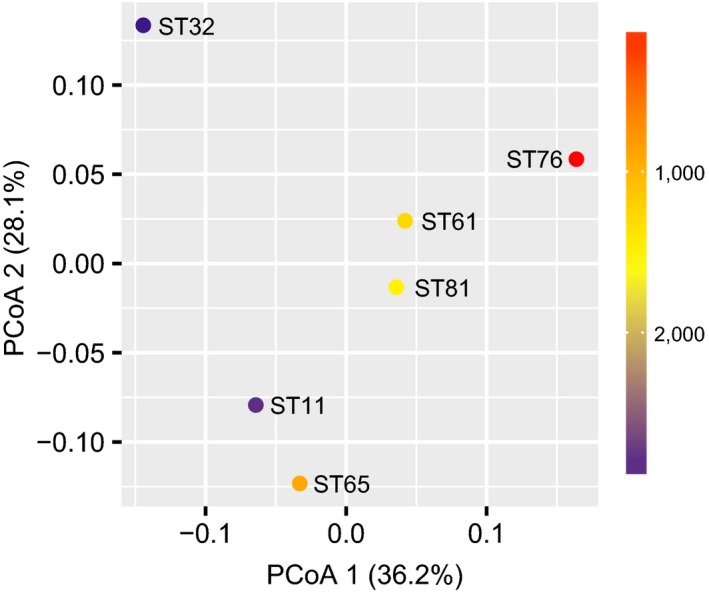
Principal coordinates analysis (PCoA) plots of the weighted UniFrac distance coupled with the water depth at each station (color coded in the heat map legend)

Principal coordinates analysis plots using UniFrac dissimilarities showed that protist communities from different water depths were well separated (Figure 5), which suggested that water depth played an important role in shaping the benthic protist communities. Specifically, a linear regression using water depths and PCoA 1 values was significant and yielded an *r* ^2^ statistic of 0.77 (Pearson's coefficient, *p* < 0.05). This outcome that water depth shaped beta diversity was also supported by the result of the Mantel test, showing a significant correlation between water depths and the weighted UniFrac distances (*r* = 0.52; *p* < 0.05; permutations = 720).

The results of the null model analysis showed that species sorting, dispersal limitation, mass effects, and drift accounted for 53.3%, 33.3%, 0%, and 13.3% of protist community pairs, respectively.

## 4 DISCUSSION

### 4.1 Diversity of benthic protists

First of all, our results uncovered the dominance of Cercozoa in protist communities of the surface sediments of the SCS (Figure 3). The dominance of Cercozoa suggests distinct microbial webs in surface sediments compared with planktonic ecosystems in the SCS, where protist communities are commonly dominated by Syndiniales (in pelagic waters) (Strassert et al., 2018; Wu, Huang, Liao, & Sun, 2014) and Radiolaria and Polycystinea (in bathypelagic waters) (Xu et al., 2017). In European coasts, the prevalence of Cercozoa generated a major difference in community composition between planktonic and benthic protists (Forster et al., 2016). However, Cercozoa failed to show dominance in estuarine sediments in Sydney Harbor (Chariton, Court, Hartley, Colloff, & Hardy, 2010) and the East China Sea (Jiang, Wang, Yu, & Liu, 2016). These disagreements support the idea that deep‐sea sediments harbor different protists than coastal and shallow‐sea sediments; thus, water depth can strongly influence benthic protist communities (Gong et al., 2015). However, it remains unclear whether the primer pair used in this study targeting the V1–V2 region, rather than the most often V4 and V9 regions, biases protist community patterns, which imposes potential effects on the dominance of Cercozoa.

A number of other groups, in addition to Cercozoa, made considerable contributions to the protist communities (Figure 3). Fungi are crucial players among marine benthos (Pasulka et al., 2016). Fungal species thrive and exhibit metabolic activities in subsurface sediments from the Peru Margin and the Peru Trench (Edgcomb, Beaudoin, Gast, Biddle, & Teske, 2011). Interestingly, some photosynthetic groups (e.g., Bacillariophyceae, Haptophyta, Prasinophyceae, and Dinophyceae) have been detected in benthic environments. Ubiquitous healthy Bacillariophyceae were recently reported in the deep sea (Agusti et al., 2015), where they may survive in resting stages (Piredda et al., 2017). Again, since DNA signatures were used in this study, we cannot rule out the possibility that these species were from the upper waters (Capo, Debroas, Arnaud, & Domaizon, 2015). Some studies based on rRNA sequencing confirm the existence of active protists in marine sediments (Bernhard et al., 2014). For example, Bacillariophyceae rRNA sequences can even be detected in subsurface sediments, suggesting that rRNA may be more stable than previously considered in benthic environments (Orsi, Biddle, & Edgcomb, 2013). In addition, Haptophyta and Prasinophyceae in fjord sediments germinate, indicating their long‐term survival in a resting stage in up to 40‐year‐old sediment layers (Ellegaard, Moestrup, Joest Andersen, & Lundholm, 2016). Haptophyta species with metabolic activity were also detected in surface sediments of the Black Sea (Coolen & Shtereva, 2009). Similarly, Dinophyceae cysts can even be germinated from 100‐year‐old sediment archives from the northern Baltic Sea (Kremp, Hinners, Klais, Leppänen, & Kallio, 2018).

### 4.2 Cercozoa dominate benthic diversity

This study detected a large number of Cercozoa OTUs (Figure 4), indicating a high diversity of ecological functions of Cercozoa (Bass & Cavalier‐Smith, 2004). For example, OTU262 is closely related to the predatory vampire amoebae (Berney et al., 2013), showing a similarity of 96% (Table A1). Several Ascetosporea OTUs are affiliated with 5 taxa with parasitical life styles (Sierra et al., 2016). Within these 5 taxa, the *Paradinium poucheti* isolate PaOi30 was isolated from the copepod host *Oithona similis* (Skovgaard & Daugbjerg, 2008) and the spot prawn *Pandalus platyceros* (Reece, Siddall, Stokes, & Burreson, 2004). In addition, several OTUs (e.g., OTU1648 and OTU1945) are closely related to a parasitical Cercozoan amoeba sp. (ex *Porphyra yezoensis*) belonging to the unknown Filosa Group III.

Notably, the top hits of Cercozoa OTUs originated from diverse habitats (Table A1), indicating that marine sediments are an outstanding reservoir of life. The majority of the OTUs were affiliated with taxa derived from benthos. For example, these representative taxa included A17 (unknown Filosa Group II) from the low‐tide sand of Vancouver Island (Bass & Cavalier‐Smith, 2004), RM1‐SGM42 (Chlorarachnea) from deep‐sea cold seep sediments (Takishita, Kakizoe, Yoshida, & Maruyama, 2010), JLJ‐11‐18 (Cercomonadida) from urban surface sediments, and NAMAKO‐14 (Euglyphida) from anoxic sediments (Takishita et al., 2007). Moreover, a set of planktonic species were included in the closest taxa, such as NS371B38 (Ascetosporea) from the 100 m water depth of the SCS (Yuan et al., 2004), BS15_B5 (unknown Filosa Group II) from the 2,593 m water depth surrounding chimneys (Sauvadet, Gobet, & Guillou, 2010), 1802E03 (Thaumatomonadida) from coastal water (Newbold et al., 2012), and RS1E4C03 (Novel Clade 2) from the Arraial do Cabo upwelling (Cury et al., 2011). These results support the idea that DNA from planktonic protists can be detected in marine sediments (Capo et al., 2015). Remarkably, unknown Filosa Group III is characterized by some taxa that were originally detected in forest soil (18s‐234) and anoxic slurries of an agricultural soil (125 T0h) (Chatzinotas, Schellenberger, Glaser, & Kolb, 2013). In contrast, unknown Filosa Group IV contains taxa from freshwaters, for example, KRL01E2 from Karla Lake, Greece (Oikonomou, Katsiapi, Karayanni, Moustaka‐Gouni, & Kormas, 2012), and MPE2‐30 from Hotoke‐Ike Lake, Antarctica (Nakai et al., 2012). The complexity of these closest retrieves indicates the existence of many potentially novel groups of protists in marine sediments.

### 4.3 Community assembly of benthic protists

Protist communities in the basin‐wide surface sediments of the SCS are mainly shaped by species sorting and dispersal limitation. This finding supports the idea that species sorting and dispersal limitation are the two key drivers of microbial community assembly in marine sediments (Petro et al., 2017). Moreover, the relative importance of species sorting indicates that benthic habitats are strongly different. Water depth may act as an important factor shaping benthic protist communities. The relationship between community dissimilarity and water depth agrees with the so‐called depth decay in marine sediments (Jacob et al., 2013). However, it should be noted that water depth may have been a proxy of a set of associated environmental variables that were unmeasured in this study. That is, benthic protist communities may be structured by something other than the water depth itself. Marine sediments represent extreme energy‐limited habitats in which species sorting can predominantly assemble benthic communities (Starnawski et al., 2017). In addition to abiotic conditions, biotic interactions can also influence benthic protist communities (i.e., top‐down controls). For example, benthic protists can impose significant grazing effects on bacterial community patterns and further influence hydrocarbon‐degrading processes in marine sediments (Beaudoin et al., 2016). This kind of driving force contributes to the relative importance of species sorting in protist communities because bacterial communities are also under selective pressure from local environments. However, the resting stage of some groups, such as Bacillariophyceae (Piredda et al., 2017), Haptophyta, Prasinophyceae (Ellegaard et al., 2016), and Dinophyceae (Kremp et al., 2018), may weaken species sorting because dormant taxa respond weakly to local environmental conditions.

The relative importance of dispersal limitation suggests that slow deep‐sea circulations (Wang, Xie, Qu, & Huang, 2011) contribute little to the dispersal of protists but generate an ecological barrier. It has been reported that benthic bacteria can show steeper distance‐decay curves than both surface‐sea and deep‐sea bacteria can (Zinger, Boetius, & Ramette, 2014). This difference may mainly result from the difference in the extent of dispersal potential of microorganisms between benthic and planktonic habitats. In contrast, Chen et al. (2017) showed that protist communities in intertidal sediments were strongly governed by spatial processes, potentially because the passive dispersal of protists contributed by water currents is very intense (i.e., mass effects) in shallow sediments relative to deep‐sea sediments. Again, disentangling protist communities can be obscured by the limitation that sedimentary DNA may be from numerous planktonic groups (Capo et al., 2015) that are not part of the indigenous and active protist community.

## 5 CONCLUSION

Our results provide baseline information on the diversity and community assembly of benthic protists in the subtropical‐tropical SCS. We show that the highly diverse Cercozoa group dominates the protist communities at the basin scale, and species sorting and dispersal limitation represent the two main forces that drive the community assembly of the benthic protists. Finally, we propose that more efforts, such as RNA‐based surveys, are needed to unveil the hidden diversity and function of protists in marine sediments.

## CONFLICT OF INTERESTS

The authors declare no conflict of interest.

## AUTHOR CONTRIBUTIONS

W.W. and B.H. conceived the study. W.W. collected sediment samples and conducted molecular laboratory work. W.W. and B.H. contributed to the data interpretation and the writing of the manuscript.

## ETHICS STATEMENT

None required.

## Data Availability

The raw sequence data were deposited in the Sequence Read Archive (NCBI) under the accession number SRP083955.

## APPENDIX 1

**Table A1:** Summary of operational taxonomic unit (OTU; 97% similarity level) assignments of the benthic Cercozoa recovered in this study. For each OTU, we provide the closest relative in GenBank with the accession number (20‐Mar‐2016 database), sequence percent similarity, BLAST score, query/subject ratio, relative abundance (%) in each sample, and taxonomy

Clone	Closest relative	GenBank Accession	Similarity (%)	BLAST score	Query/Subject	ST11	ST32	ST61	ST65	ST76	ST81	Taxonomy
OTU1102	Uncultured eukaryote band JLJ‐8‐41	JN846845	93	540	343/368	0.29	0.16	0.06	0.42	0.10	0.22	Aconchulinida
OTU1382	Uncultured marine cercozoan clone BS15_B5	FN598356	92	527	343/371	0.01	0.00	0.00	0.01	0.00	0.00	Aconchulinida
OTU1713	Uncultured marine cercozoan clone BS15_B5	FN598356	91	503	337/369	0.04	0.00	0.00	0.00	0.02	0.00	Aconchulinida
OTU1958	Uncultured marine cercozoan clone BS15_B5	FN598356	93	536	343/369	0.02	0.00	0.00	0.19	0.17	0.00	Aconchulinida
OTU235	Uncultured Rhizaria clone F.13_2073	KP685352	90	473	335/371	0.00	0.02	0.45	0.05	0.00	0.00	Aconchulinida
OTU262	Vampyrellida sp. KibAr	KC779519	96	604	355/368	0.00	0.00	0.31	0.01	0.00	0.23	Aconchulinida
OTU1091	Uncultured eukaryote band MS_euk3_18	FJ785960	90	477	331/366	0.41	0.19	0.04	0.01	0.00	0.17	Ascetosporea
OTU1110	Uncultured eukaryote band MS_euk3_18	FJ785960	91	490	333/365	0.00	0.00	0.00	0.25	0.00	0.01	Ascetosporea
OTU1156	Haplosporidian parasite of Pandalus platyceros clone 3	AY449715	96	597	350/363	2.52	3.27	0.33	0.88	0.03	2.64	Ascetosporea
OTU1161	Uncultured eukaryote band MS_euk3_18	FJ785960	92	508	339/369	0.00	0.00	0.00	1.50	0.00	0.01	Ascetosporea
OTU1187	Uncultured eukaryote band MS_euk3_18	FJ785960	94	555	344/365	0.35	0.64	0.00	0.21	0.00	0.01	Ascetosporea
OTU1244	Uncultured eukaryote clone 1084_ts10water_13709	KJ003964	90	459	328/366	0.11	1.73	0.09	2.64	0.00	0.06	Ascetosporea
OTU1247	Haplosporidian parasite of Pandalus platyceros clone 3	AY449715	94	542	340/363	2.89	2.51	0.40	1.97	1.00	2.64	Ascetosporea
OTU125	Uncultured eukaryote band MS_euk3_18	FJ785960	93	532	340/365	0.01	0.00	0.00	0.08	0.00	0.00	Ascetosporea
OTU1278	Uncultured eukaryote band MS_euk3_18	FJ785960	91	484	333/366	0.00	0.02	0.02	0.00	0.00	0.00	Ascetosporea
OTU128	Uncultured eukaryote clone 1084_ts10water_13709	KJ003964	91	484	329/362	0.08	0.01	0.01	0.10	0.00	0.01	Ascetosporea
OTU134	Uncultured eukaryote band MS_euk3_18	FJ785960	91	494	335/367	0.07	0.19	0.07	0.03	0.00	0.00	Ascetosporea
OTU1513	Uncultured eukaryote band MS_euk3_18	FJ785960	91	486	332/365	0.52	0.34	2.61	0.01	0.00	0.07	Ascetosporea
OTU1514	Haplosporidian parasite of Pandalus platyceros clone 3	AY449715	93	529	338/363	0.04	0.04	0.01	0.00	0.00	0.03	Ascetosporea
OTU1551	Paradinium poucheti isolate PaOi30	EU189031	92	518	343/373	0.32	0.00	0.00	0.01	0.00	0.00	Ascetosporea
OTU1581	Haplosporidian parasite of Pandalus platyceros clone 3	AY449715	94	553	342/363	0.05	0.09	0.00	0.00	0.00	0.00	Ascetosporea
OTU1610	Haplosporidian parasite of Pandalus platyceros clone 3	AY449715	93	520	336/363	0.05	0.00	0.01	0.00	0.00	0.00	Ascetosporea
OTU1616	Uncultured eukaryote band MS_euk3_18	FJ785960	90	477	332/367	0.03	2.65	1.11	0.32	9.63	0.01	Ascetosporea
OTU1625	Uncultured eukaryote band MS_euk3_18	FJ785960	92	514	337/365	0.02	0.07	0.00	0.00	0.00	0.00	Ascetosporea
OTU1751	Haplosporidian parasite of Pandalus platyceros clone 3	AY449715	94	553	342/363	0.09	0.19	0.02	0.07	0.00	0.30	Ascetosporea
OTU1758	Uncultured eukaryote clone 1084_ts10water_13709	KJ003964	90	466	327/363	0.02	0.01	0.01	0.00	0.00	0.00	Ascetosporea
OTU1807	Haplosporidian parasite of Pandalus platyceros clone 3	AY449715	91	484	332/366	0.00	0.01	0.00	0.05	0.00	0.00	Ascetosporea
OTU1814	Uncultured marine eukaryote clone NS371B38	AJ829787	95	577	354/374	0.00	0.03	0.01	0.01	0.00	0.00	Ascetosporea
OTU1817	Uncultured eukaryote band MS_euk3_18	FJ785960	93	525	339/365	0.00	0.01	0.09	0.00	0.00	0.00	Ascetosporea
OTU1863	Uncultured eukaryote clone 1084_ts10water_13709	KJ003964	91	490	333/365	0.00	0.01	0.00	0.04	0.00	0.00	Ascetosporea
OTU1882	Uncultured eukaryote band MS_euk3_18	FJ785960	90	475	331/366	0.00	0.00	0.00	0.41	0.00	0.00	Ascetosporea
OTU1923	Haplosporidian parasite of Pandalus platyceros clone 3	AY449715	95	571	347/365	0.46	0.24	0.32	0.28	0.00	0.23	Ascetosporea
OTU1943	Uncultured eukaryote band MS_euk3_18	FJ785960	93	532	342/367	0.32	0.17	0.00	0.27	0.00	0.01	Ascetosporea
OTU1976	Uncultured eukaryote band MS_euk3_18	FJ785960	93	536	342/367	0.00	0.01	0.00	0.04	0.00	0.00	Ascetosporea
OTU198	Uncultured eukaryote band MS_euk3_18	FJ785960	94	549	343/365	0.00	0.08	0.00	0.07	0.00	0.75	Ascetosporea
OTU1980	Uncultured marine eukaryote clone NS371B38	AJ829787	98	636	364/373	0.10	0.94	0.39	0.29	0.07	0.13	Ascetosporea
OTU2018	Haplosporidian parasite of Pandalus platyceros clone 3	AY449715	91	497	332/363	0.12	0.01	0.00	0.04	0.00	0.00	Ascetosporea
OTU2032	Uncultured marine eukaryote clone NS371B38	AJ829787	95	584	353/371	0.00	0.02	0.00	0.02	0.00	0.00	Ascetosporea
OTU2033	Uncultured eukaryote band MS_euk3_18	FJ785960	97	619	355/365	0.02	0.00	0.00	0.00	0.00	0.00	Ascetosporea
OTU223	Uncultured eukaryote band MS_euk3_18	FJ785960	93	529	339/365	0.00	0.01	0.01	0.07	0.00	0.00	Ascetosporea
OTU258	Uncultured eukaryote band MS_euk3_18	FJ785960	92	503	335/365	0.09	0.00	0.01	0.00	0.00	0.00	Ascetosporea
OTU261	Uncultured eukaryote band MS_euk3_18	FJ785960	89	451	326/365	0.00	0.00	0.11	1.94	0.00	0.05	Ascetosporea
OTU374	Haplosporidian parasite of Pandalus platyceros clone 3	AY449715	94	547	341/363	0.25	0.02	0.00	0.07	0.00	0.00	Ascetosporea
OTU38	Haplosporidian parasite of Pandalus platyceros clone 3	AY449715	93	538	339/363	0.00	0.00	0.00	0.01	0.00	0.19	Ascetosporea
OTU399	Uncultured eukaryote band MS_euk3_18	FJ785960	90	473	332/368	0.00	0.00	0.01	0.20	0.09	0.07	Ascetosporea
OTU484	Uncultured eukaryote band MS_euk3_18	FJ785960	92	497	334/365	0.04	0.00	0.00	0.03	0.00	0.00	Ascetosporea
OTU503	Paradinium poucheti isolate PaOi30	EU189031	91	507	341/373	0.04	0.00	0.00	0.13	0.03	0.10	Ascetosporea
OTU540	Uncultured eukaryote band MS_euk3_18	FJ785960	93	531	340/365	0.00	0.06	0.02	0.03	0.00	0.00	Ascetosporea
OTU549	Haplosporidian parasite of Pandalus platyceros clone 3	AY449715	91	481	337/372	0.29	0.12	0.00	0.13	0.00	0.07	Ascetosporea
OTU611	Uncultured eukaryote clone 1084_ts10water_13709	KJ003964	90	468	328/364	0.01	0.03	0.00	0.00	0.00	0.04	Ascetosporea
OTU702	Haplosporidian parasite of Pandalus platyceros clone 3	AY449715	93	520	338/365	0.01	0.16	0.00	0.02	0.00	0.00	Ascetosporea
OTU709	Haplosporidian parasite of Pandalus platyceros clone 3	AY449715	94	542	340/363	0.00	0.06	0.06	0.06	0.00	0.00	Ascetosporea
OTU768	Haplosporidian parasite of Pandalus platyceros clone 3	AY449715	94	544	341/364	0.05	0.04	0.04	0.01	0.00	0.00	Ascetosporea
OTU802	Uncultured eukaryote band MS_euk3_18	FJ785960	95	569	347/365	0.01	0.08	0.00	0.10	0.02	0.08	Ascetosporea
OTU811	Haplosporidian parasite of Pandalus platyceros clone 3	AY449715	90	466	328/365	0.00	4.70	0.19	0.12	0.00	0.00	Ascetosporea
OTU844	Uncultured eukaryote band MS_euk3_18	FJ785960	93	540	344/368	0.01	0.08	0.00	0.01	0.00	0.00	Ascetosporea
OTU886	Uncultured eukaryote band MS_euk3_18	FJ785960	92	516	338/366	0.26	3.10	0.64	0.28	0.00	0.41	Ascetosporea
OTU891	Haplosporidian parasite of Pandalus platyceros clone 3	AY449715	91	497	332/363	0.00	0.20	0.02	0.02	0.00	0.02	Ascetosporea
OTU950	Haplosporidian parasite of Pandalus platyceros clone 3	AY449715	94	558	343/363	0.10	0.01	0.02	0.00	0.00	0.00	Ascetosporea
OTU965	Haplosporidian parasite of Pandalus platyceros clone 3	AY449715	91	492	331/363	0.02	0.08	0.04	0.00	0.00	0.00	Ascetosporea
OTU1022	Uncultured marine eukaryote clone I‐8‐MC832‐OTU‐44	KC771152	94	564	348/369	0.00	0.00	0.13	0.09	0.07	0.00	Cercomonadida
OTU1366	Uncultured eukaryote clone CYSGM‐14	AB275097	96	599	352/365	0.00	0.03	0.14	0.00	0.03	0.00	Cercomonadida
OTU1422	Uncultured eukaryote band JLJ‐11‐18	JN846847	97	627	360/370	0.00	0.15	0.18	0.00	0.07	0.00	Cercomonadida
OTU1671	Uncultured marine picoeukaryote clone 1802000	FR874390	95	575	351/370	0.01	0.00	1.16	0.00	0.24	0.00	Cercomonadida
OTU1241	Uncultured eukaryote clone 18s2‐34	EU798716	95	571	351/371	0.03	0.00	0.00	0.00	0.05	0.00	Chlorarachnea
OTU1386	Uncultured eukaryote clone RM2‐SGM59	AB505567	99	662	362/364	0.07	0.09	0.03	0.02	0.03	0.30	Chlorarachnea
OTU1584	Uncultured eukaryote clone ST8900.004	KF130513	98	617	346/352	0.00	0.08	0.01	0.07	0.00	0.00	Chlorarachnea
OTU240	Uncultured eukaryote clone T0h‐125	KF357281	92	521	341/370	0.00	0.00	0.00	0.05	0.02	0.00	Chlorarachnea
OTU327	Lotharella globosa strain LEX01	JF826444	91	473	321/352	0.07	0.20	0.09	1.14	0.00	0.56	Chlorarachnea
OTU877	Uncultured eukaryote clone RM1‐SGM42	AB505499	92	521	351/381	0.04	0.11	0.09	0.00	0.00	0.00	Chlorarachnea
OTU1038	Uncultured eukaryote clone Seq‐16_C23	KC336998	99	662	360/361	0.01	0.00	0.13	0.29	1.00	0.28	Euglyphida
OTU1113	Uncultured eukaryote clone Seq‐16_C23	KC336998	96	593	349/362	0.00	0.00	0.00	0.01	0.17	0.00	Euglyphida
OTU1129	Uncultured cercozoan clone 12‐4.1	AY620337	92	514	343/372	0.00	0.03	0.04	0.00	0.00	0.00	Euglyphida
OTU1131	Uncultured cercozoan clone 12‐4.1	AY620337	94	568	352/373	0.00	0.04	0.00	0.00	0.00	0.04	Euglyphida
OTU1183	Uncultured eukaryote clone S2‐44	KJ568924	91	486	336/370	0.00	0.01	0.00	0.00	0.16	0.00	Euglyphida
OTU1283	Uncultured eukaryote clone NAMAKO‐14	AB252754	99	652	353/356	0.00	0.01	0.00	0.12	0.02	0.18	Euglyphida
OTU1300	Uncultured cercozoan clone F.18_395	KP685356	93	538	342/366	0.00	0.03	0.02	0.00	0.00	0.00	Euglyphida
OTU1312	Uncultured eukaryote clone MS605‐58	HM369676	92	523	341/369	0.08	0.00	0.00	0.00	0.02	0.00	Euglyphida
OTU1330	Uncultured eukaryote clone X6‐84	KJ568694	99	638	355/360	0.05	0.17	0.00	0.01	0.00	0.00	Euglyphida
OTU148	Uncultured eukaryote clone 18S‐AK‐B‐46	AB238153	93	532	342/368	0.00	0.06	0.02	0.00	0.00	0.00	Euglyphida
OTU1499	Cercomonadida clone D0570_57_S	EU646942	96	595	355/370	0.00	0.00	0.00	0.03	0.10	0.00	Euglyphida
OTU1569	Uncultured cercozoan clone 7‐5.4	AY620332	95	580	350/368	0.01	0.00	1.72	0.00	0.02	0.00	Euglyphida
OTU1642	Uncultured cercozoan clone 7‐1.3	AY620333	91	492	337/370	0.02	0.00	0.00	0.00	0.02	0.00	Euglyphida
OTU1665	Uncultured cercozoan clone F.18_473	KP685355	94	556	350/373	0.07	0.00	0.01	0.00	0.02	0.00	Euglyphida
OTU1760	Uncultured cercozoan clone 12‐4.1	AY620337	99	675	366/367	0.00	0.00	0.00	0.01	0.47	0.00	Euglyphida
OTU180	Uncultured cercozoan clone F.13_4422	KP685343	92	503	339/370	0.00	0.00	0.08	0.00	0.02	0.00	Euglyphida
OTU1885	Uncultured eukaryote clone F6‐47	KJ568839	96	593	535/368	0.00	0.00	0.00	0.00	0.02	0.11	Euglyphida
OTU2014	Uncultured cercozoan clone DH1321F11	KM067448	91	486	334/369	0.00	0.01	0.03	0.00	0.00	0.00	Euglyphida
OTU3	Uncultured eukaryote clone X6‐001	KF378517	91	486	338/373	0.23	0.19	0.31	0.00	0.00	0.00	Euglyphida
OTU412	Uncultured cercozoan clone 7‐5.4	AY620332	98	628	359/368	0.40	0.00	0.48	0.00	0.07	0.00	Euglyphida
OTU438	Uncultured marine picoeukaryote clone 1804G02	FR874476	95	568	348/368	0.51	0.01	0.00	0.00	0.00	0.00	Euglyphida
OTU580	Uncultured eukaryote clone G1029.0046A46	KP143011	92	518	340/369	0.03	0.00	0.00	0.00	0.02	0.00	Euglyphida
OTU71	Uncultured eukaryote clone RS1E1F01	HM227764	99	643	358/363	0.00	0.00	0.00	0.00	0.07	0.10	Euglyphida
OTU778	Uncultured cercozoan clone F.18_473	KP685355	94	564	351/372	0.00	0.01	0.24	0.00	0.00	0.00	Euglyphida
OTU839	Uncultured cercozoan clone 13‐2.2	AY620297	92	508	339/369	0.00	0.01	0.07	0.00	0.02	0.00	Euglyphida
OTU863	Uncultured cercozoan clone F.18_395	KP685356	94	547	346/369	0.90	0.02	1.05	0.44	0.17	1.02	Euglyphida
OTU1323	Uncultured cercozoan clone F.18_117	KP685366	99	673	366/367	0.01	0.00	0.00	0.00	0.41	0.00	Noveb Clade 2 (Imbricatea)
OTU1494	Uncultured eukaryote clone G1029.0046A46	KP143011	94	562	347/368	0.00	0.00	0.00	0.09	0.21	0.00	Noveb Clade 2 (Imbricatea)
OTU150	Uncultured cercozoan clone F.18_316	KP685357	97	610	354/365	0.00	0.00	0.02	0.03	0.47	0.00	Noveb Clade 2 (Imbricatea)
OTU1688	Uncultured eukaryote clone DSGM‐55	AB275055	99	667	363/364	0.02	0.01	0.12	0.11	4.18	0.18	Noveb Clade 2 (Imbricatea)
OTU1768	Uncultured marine eukaryote clone BTQB20040719.0165	FJ221790	98	628	354/361	0.01	0.03	0.02	0.16	0.02	0.00	Noveb Clade 2 (Imbricatea)
OTU346	Uncultured eukaryote clone RS1E4C03	HM227981	92	523	343/371	0.00	0.00	0.01	0.00	0.26	0.00	Noveb Clade 2 (Imbricatea)
OTU475	Uncultured marine eukaryote clone SGB2‐151	JN585087	97	606	353/365	0.00	0.00	0.00	0.20	0.36	0.03	Noveb Clade 2 (Imbricatea)
OTU505	Uncultured marine eukaryote clone SGB2‐151	JN585087	94	544	346/370	0.01	0.00	0.00	0.00	0.00	0.23	Noveb Clade 2 (Imbricatea)
OTU513	Uncultured marine cercozoan clone RA070411N.101	FJ431323	95	568	350/370	0.00	0.00	0.00	0.03	0.12	0.00	Noveb Clade 2 (Imbricatea)
OTU573	Uncultured cercozoan clone 7‐6.6	AY620336	94	560	346/367	0.01	0.00	0.10	0.00	0.00	0.00	Noveb Clade 2 (Imbricatea)
OTU651	Uncultured eukaryote clone T9‐A‐7	KT277599	91	486	336/371	0.00	0.04	0.00	0.00	0.02	0.00	Noveb Clade 2 (Imbricatea)
OTU1120	Cercozoa sp. CC‐2009f	FJ824130	93	538	344/370	0.00	0.00	0.15	0.00	0.00	0.09	Thaumatomonadida
OTU1340	Uncultured marine picoeukaryote clone 1802E03	FR874390	96	593	354/370	0.00	0.02	0.00	0.00	0.09	0.00	Thaumatomonadida
OTU1448	Uncultured eukaryotic clone XMCC9	DQ667659	95	580	352/370	0.00	0.00	0.15	0.00	0.03	0.00	Thaumatomonadida
OTU158	Uncultured marine eukaryote clone UEPACMp4	DQ369017	96	597	356/371	0.01	0.01	0.00	0.00	2.66	0.00	Thaumatomonadida
OTU1704	Uncultured cercozoan clone 4b‐33	FN690391	98	634	363/372	2.40	1.36	2.41	2.85	0.26	5.26	Thaumatomonadida
OTU1846	Uncultured Rhizaria clone F.13_4123	KP685347	95	577	350/369	0.00	0.00	0.00	0.08	0.02	0.00	Thaumatomonadida
OTU1951	Uncultured marine picoeukaryote clone 1802E03	FR874390	96	590	353/369	0.00	0.00	0.08	0.00	0.07	0.00	Thaumatomonadida
OTU302	Uncultured marine picoeukaryote clone 1802E03	FR874390	100	682	369/369	0.18	0.01	8.73	0.33	0.31	0.18	Thaumatomonadida
OTU362	Cercomonadida clone D0570_57_S	EU646942	92	507	337/368	0.08	0.00	0.01	0.01	0.02	0.00	Thaumatomonadida
OTU369	Uncultured cercozoan clone 4b‐33	FN690391	97	621	363/375	0.08	0.01	0.00	0.02	0.02	0.02	Thaumatomonadida
OTU490	Uncultured marine eukaryote clone ME_Euk_FW1	GU385595	97	608	356/368	0.01	0.00	0.10	0.01	1.52	0.00	Thaumatomonadida
OTU637	Cercozoa sp. CC‐2009f	FJ824130	93	544	346/371	0.00	0.00	1.83	0.00	0.05	0.00	Thaumatomonadida
OTU1016	Uncultured eukaryote clone RM1‐SGM44	AB505501	96	595	351/365	0.00	0.04	0.00	0.00	0.03	0.00	Thecofilosea
OTU1322	Uncultured Rhizaria clone F.18_375	KP685362	99	658	365/369	0.60	1.25	3.62	1.15	9.65	7.87	Thecofilosea
OTU136	Uncultured eukaryote band MS_euk3_17	FJ785961	96	604	356/370	0.00	0.00	0.00	0.12	0.07	0.00	Thecofilosea
OTU1362	Uncultured cercozoan clone 552	HQ696567	93	534	344/370	0.00	0.00	0.04	0.06	0.00	0.00	Thecofilosea
OTU139	Uncultured cercozoan clone 6c‐D8	FN690359	99	658	364/368	0.11	0.04	0.18	0.01	0.35	0.08	Thecofilosea
OTU1628	Uncultured eukaryote clone 8b11	KJ925997	98	649	363/369	0.00	0.00	0.16	0.00	0.03	0.00	Thecofilosea
OTU168	Uncultured eukaryote clone SS1_E_01_26	EU050975	98	630	359/368	0.00	0.00	0.01	0.00	0.02	0.00	Thecofilosea
OTU1726	Uncultured eukaryote clone BB01_73	AY885063	98	651	366/372	0.00	0.00	0.00	0.01	0.05	0.00	Thecofilosea
OTU1929	Uncultured cercozoan clone 7‐5.5	AY620347	97	617	359/371	0.08	0.01	0.00	0.04	0.02	0.00	Thecofilosea
OTU280	Cercozoa sp. CC‐2009c	FJ824127	98	638	363/371	0.00	0.00	0.16	0.06	0.05	0.07	Thecofilosea
OTU289	Uncultured cercozoan clone A14	AY620321	97	621	358/369	0.00	0.00	0.03	0.00	0.02	0.00	Thecofilosea
OTU622	Uncultured eukaryote clone SS1_E_01_26	EU050975	99	652	364/369	1.06	1.66	13.25	2.53	2.33	15.02	Thecofilosea
OTU756	Uncultured cercozoan clone 6b‐F6	FN690389	98	640	364/372	0.00	0.00	0.02	0.04	0.07	0.00	Thecofilosea
OTU799	Uncultured eukaryote band MS_euk3_17	FJ785961	98	643	362/369	0.00	0.00	0.04	0.07	0.29	0.00	Thecofilosea
OTU803	Uncultured cercozoan clone 12‐4.4	AY620350	97	621	359/370	0.01	0.00	0.02	0.16	0.03	0.12	Thecofilosea
OTU852	Uncultured eukaryote clone SS1_E_02_29	EU050977	99	656	366/371	0.01	0.01	0.00	0.00	0.14	0.00	Thecofilosea
OTU912	Uncultured Rhizaria clone F.13_4123	KP685347	97	614	357/369	0.00	0.00	0.00	0.14	0.02	0.00	Thecofilosea
OTU1575	Uncultured cercozoan clone BS15_B5	FN598356	92	516	340/370	0.00	0.01	0.03	0.20	0.00	0.00	Unknown Filosa Group I
OTU1906	Uncultured microeukaryote clone ME‐19	KC851800	99	665	364/366	0.07	0.06	0.03	0.07	0.19	0.20	Unknown Filosa Group I
OTU244	Uncultured eukaryote clone 13	KP187808	95	580	351/369	0.00	0.00	0.00	0.07	0.00	0.00	Unknown Filosa Group I
OTU333	Uncultured marine cercozoan clone BS15_B5	FN598356	92	525	345/374	0.26	0.10	0.05	0.02	0.02	0.06	Unknown Filosa Group I
OTU421	Uncultured eukaryote clone 13	KP187808	91	505	345/378	0.01	0.00	0.03	0.04	0.00	0.00	Unknown Filosa Group I
OTU616	Uncultured marine cercozoan clone BS15_B5	FN598356	95	586	353/370	0.00	0.00	0.05	0.07	0.02	0.00	Unknown Filosa Group I
OTU857	Uncultured eukaryote clone RM2‐SGM59	AB505567	92	505	339/370	0.11	0.00	0.02	0.00	0.00	0.22	Unknown Filosa Group I
OTU93	Uncultured marine cercozoan clone BS15_B5	FN598356	92	514	341/371	0.12	0.02	0.01	0.00	0.00	0.15	Unknown Filosa Group I
OTU1578	Uncultured cercozoan clone A17	AY620324	93	534	346/374	0.01	0.04	0.02	0.02	0.14	0.00	Unknown Filosa Group II
OTU219	Uncultured eukaryote clone QZ.18S_1	KT201564	97	627	362/373	0.00	0.00	0.00	0.02	0.00	0.04	Unknown Filosa Group II
OTU569	Uncultured cercozoan clone A17	AY620324	94	571	352/373	0.03	0.00	0.16	0.01	0.00	0.06	Unknown Filosa Group II
OTU11	Uncultured eukaryote clone RM2‐SGM64	AB505572	99	656	362/365	0.00	0.00	0.00	0.01	0.03	0.10	Unknown Filosa Group III
OTU1372	Uncultured eukaryote clone T0h‐125	KF357281	92	514	342/373	0.00	0.00	0.01	0.00	0.35	0.00	Unknown Filosa Group III
OTU1373	Uncultured marine cercozoan clone BS15_B5	FN598356	94	566	349/370	0.00	0.00	0.00	0.02	0.21	0.01	Unknown Filosa Group III
OTU1648	Cercozoan amoeba sp. ex Porpyra yezoensis	DQ666485	90	490	341/377	0.00	0.00	0.00	0.03	0.10	0.00	Unknown Filosa Group III
OTU1826	Uncultured marine cercozoan clone BS15_B5	FN598356	94	566	350/371	0.24	0.09	0.06	0.29	0.16	0.44	Unknown Filosa Group III
OTU1944	Uncultured cercozoan clone 4‐1.8	AY620267	96	593	355/371	0.00	0.00	0.03	0.08	0.03	0.06	Unknown Filosa Group III
OTU1945	Cercozoan amoeba sp. ex Porpyra yezoensis	DQ666485	90	486	340/376	0.03	0.00	0.04	0.11	0.28	0.00	Unknown Filosa Group III
OTU442	Uncultured cercozoan clone 4‐1.8	AY620267	90	475	334/371	0.00	0.02	0.00	0.00	0.02	0.00	Unknown Filosa Group III
OTU45	Uncultured eukaryote clone BF‐A4‐1‐12c	EU860499	95	573	351/371	0.00	0.01	0.00	0.10	0.05	0.00	Unknown Filosa Group III
OTU658	Uncultured eukaryote clone 125	KF357281	90	486	337/373	0.00	0.00	0.00	0.03	0.00	0.11	Unknown Filosa Group III
OTU884	Uncultured cercozoan clone 7‐5.2	AY620346	95	588	353/370	0.00	0.00	0.01	0.00	0.03	0.00	Unknown Filosa Group III
OTU902	Uncultured eukaryote clone T0h‐125	KF357281	96	612	357/370	0.01	0.01	0.00	0.03	0.02	0.01	Unknown Filosa Group III
OTU1299	Uncultured marine eukaryote clone NA2_1B8	EF526891	98	652	366/372	0.44	0.24	0.63	0.00	0.50	0.00	Unknown Filosa Group IV
OTU1572	Uncultured eukaryotic clone CYSGM‐17	AB275100	99	649	362/367	0.01	0.35	0.00	0.01	0.17	0.00	Unknown Filosa Group IV
OTU41	Uncultured marine eukaryote clone ME_Euk_FW9	GU385688	97	625	359/369	0.25	0.00	0.01	0.02	0.02	0.00	Unknown Filosa Group IV
OTU538	Uncultured eukaryote clone MPE2‐30	AB695524	95	586	356/374	0.00	0.00	0.00	0.02	0.03	0.00	Unknown Filosa Group IV
OTU823	Uncultured Rhizaria clone F.13_2119	KP685342	96	608	357/371	0.00	0.00	0.08	0.00	0.07	0.00	Unknown Filosa Group IV
OTU952	Uncultured eukaryote clone T0h‐81	KF357374	94	558	349/371	0.04	0.01	0.00	0.07	0.00	0.00	Unknown Filosa Group IV
OTU1001	Cercozoa sp. COHH 48	GU320591	91	486	336/371	0.00	0.00	0.06	0.00	0.05	0.00	Unknown Endomyxa Group I
OTU1409	Uncultured marine cercozoan clone BS11_B2	FN598314	99	676	370/372	0.20	0.76	0.24	0.35	0.02	0.16	Unknown Endomyxa Group I
OTU1749	Uncultured marine cercozoan clone BS11_B2	FN598314	93	542	346/372	0.54	0.39	0.08	0.76	0.00	0.32	Unknown Endomyxa Group I
OTU1781	Uncultured marine cercozoan clone BS15_B5	FN598356	91	503	338/370	0.00	0.09	0.00	0.00	0.02	0.00	Unknown Endomyxa Group I
OTU1841	Uncultured eukaryote clone SGYH927	KJ762794	99	675	371/374	0.07	0.13	0.05	0.00	0.00	0.06	Unknown Endomyxa Group I
OTU958	Uncultured marine cercozoan clone BS15_B5	FN598356	92	521	347/377	0.04	0.00	0.00	0.00	0.05	0.00	Unknown Endomyxa Group I
OTU1775	Uncultured eukaryote clone SGYY525	KJ757438	97	612	356/368	1.56	7.45	2.31	4.08	4.45	2.74	Unknown Endomyxa Group II
OTU329	Uncultured marine cercozoan clone BS15_B5	FN598356	89	464	332/371	1.87	3.58	1.40	0.61	0.07	2.29	Unknown Endomyxa Group II
OTU1336	Uncultured marine cercozoan clone BS15_B5	FN598356	93	540	344/369	0.01	0.00	0.02	0.01	0.16	0.00	Unknown Cercozoa
OTU1549	Uncultured eukaryote band JLJ‐8‐41	JN846845	94	549	345/368	0.00	0.00	0.00	0.23	0.36	0.13	Unknown Cercozoa
OTU1622	Uncultured cercozoan clone 7‐6.1	AY620355	93	538	344/369	0.00	0.00	0.02	0.05	0.05	0.00	Unknown Cercozoa
OTU1910	Uncultured eukaryote clone KRL01E2	JN090862	94	555	351/375	0.00	0.00	0.07	0.00	0.02	0.00	Unknown Cercozoa
OTU440	Uncultured microeukaryote clone ME‐12	KC851793	96	599	355/370	0.00	0.08	0.12	0.00	0.22	0.00	Unknown Cercozoa
OTU488	Uncultured eukaryote band JLJ‐8‐41	JN846845	96	586	352/368	0.00	0.00	0.00	0.05	0.62	0.00	Unknown Cercozoa
OTU621	Uncultured eukaryote clone F6‐39	KJ568831	95	580	354/373	0.00	0.00	0.00	0.01	0.14	0.00	Unknown Cercozoa
